# Molecular cloning and functional verification of chalcone synthase genes from cassava (*Manihot esculenta* Crantz) in defense against *Tetranychus cinnabarinus* infestation

**DOI:** 10.1371/journal.pone.0321276

**Published:** 2025-04-24

**Authors:** Yanni Yang, Kaiwen Zheng, Limei Gao, Yakang Hu, Cuixia Liu, Fei Li, Ming Liu

**Affiliations:** 1 Guangxi Key Laboratory of Plant Conservation and Restoration Ecology in Karst Terrain, Guangxi Institute of Botany, Guangxi Zhuang Autonomous Region and Chinese Academy of Sciences, Guilin, Guangxi, China; 2 College of Agronomy, Guangxi University, Nanning, China; Guizhou University, CHINA

## Abstract

Secondary metabolites such as flavonoids play an important role in protecting plants from biological agents such as fungi, pathogens, bacteria and pests. Chalcone synthase (CHS) is the first enzyme in the plant flavonoid biosynthesis pathway, and is also a key enzyme and rate-limiting enzyme in the secondary metabolite production pathway, which has very important physiological significance in plants. Despite extensive characterization in various plants, the functions of CHS in cassava remain unknown. Here, *MeCH*S1, *MeCHS*3 and *MeCH*S5 genes from *Manihot esculenta* Crantz were isolated and functionally analyzed. The results showed that the over-expression of the three *MeCHS*s were beneficial to control the further reproduction of *Tetranychus cinnabarinus*. At the same time, the transfer of *MeCH*S1, *MeCHS*3 and *MeCH*S5 genes can promote the synthesis of more secondary metabolites in *Arabidopsis thaliana*. Heterologous expression in *A. thaliana* indicated the presence of different expression levels of the three *MeCHS*s in defense against *T. cinnabarinus* infestation. Correlation analysis showed that the expression of *MeCHS*s were positively correlated with the synthesis of secondary metabolites, and negatively correlated with survival rate of *T. cinnabarinus*. These results indicate that the different expression levels of *MeCHS* genes lead to the difference in the synthesis of secondary metabolites, and thus the resistance of *A. thaliana* to *T. cinnabarinus* is also different.

## Introduction

Cassava (*Manihot esculenta* Crantz, Euphorbiaceae) is one of the most important root tuber energy crops widely cultivated in the tropical and temperate regions such as Asia, Africa and Americas [1]. It has a high carbohydrate production potential and adaptability to a variety of environments, but is susceptible to mites, especially *Tetranychus cinnabarinus*, which is one of the most serious worldwide pest mites affecting cassava, causing severe economic losses [2].

In our previous study, we found that secondary metabolism products such as flavonoids were increased in cassava leaves infested by *T. cinnabarinus* [3]. In fact, flavonoids have been shown to have a wide range of biological functions in anthocyanin deposition, resistance to ultraviolet radiation [4–6], plant growth and development resistance, auxin transport, pollen reproduction, and so forth [7–9]. In addition, as an antimicrobial metabolite, it plays an important role in protecting plants from biological stresses [10]. Flavonoids are commonly found in the epidermal cells of plant organs such as leaves, flowers, stems, roots, seeds, and fruits. Flavonoids often exist in various chemical forms such as monomers, dimers and oligomers. At present, more than 9000 kinds of flavonoid monomers have been successfully isolated [11].

Flavonoid biosynthesis represents a critical secondary metabolic pathway in plants. The main steps of this pathway have been well-characterized, and the detailed metabolic pathway was illustrated in S1 Fig of the Supplementary Figure. According to the structural differences of the products, the metabolites mainly included the following: flavones, flavonols, anthocyanins, condensed tannins, chalcones and isoflavonoids [12,13]. The metabolites of these flavonoids are derived from the phenylalanine metabolic pathway in plants. The main catalytic enzymes involved in flavonoid biosynthesis include PAL (phenylalanine ammonia-lyase), C4H (cinnamic acid 4-hydroxylase), 4CL (4-coumarate-CoA ligase), CHS (chalcone synthase), F3’H (flavonoid 3’-hydroxylase), FLS (flavonol synthase), DFR (dihydroflavonol 4-reductase), LAR (leucoanthocyanidin reductase), LDOX (leucoanthocyanidin dioxygenase), UFGT (UDP-glucose flavonoid glucosyltransferase) and ANR (anthocyanidin reductase). In higher plants, multiple genes within this pathway have been implicated in biotic defense mechanisms, particularly in response to pathogen attacks. Notably, chalcone synthase (CHS) serves as the first key enzyme and rate-limiting step in this biosynthetic process [14]. Until recently, the role of CHS genes against *T. cinnabarinus* had not been systematically studied in cassava. In many plant species, CHS was encoded by a small family of genes with highly conserved homology both within and between species [15]. Through the comprehensive study on the CHS family of cassava, we confirmed that some *MeCHS* genes belonging to the LAP (leucine aminoptidase) subfamily played a crucial role in the mite-resistant response of *T. cinnabarinus* [16,17].

The product of the CHS reaction is a pivotal precursor for a large array of secondary metabolites derived from malonyl-CoA and p-coumaroyl-CoA. CHS catalyzes the condensation reaction of three molecules of malonyl-CoA and with one molecule of 4-coumaryl-CoA to produce the basic skeleton material of various flavonoid compounds: naringenin chalcone, which is tetrahydroxychalcon [17]. The other pathway is catalyzed by CHS to form Chalcones trihydroxychalcone, which gives the plant flowers a yellow color [18]. Therefore, the chemical reaction catalyzed by CHS lies at the junction of phenylalanine metabolic pathway and flavonoid metabolic pathway, which is also an important rate-limiting step of the whole flavonoid biosynthesis pathway. As a basic material for the synthesis of various flavonoids, chalcones are located in the upstream of the flavonoid biosynthesis pathway. When the substrate from the upstream synthesizes chalcones under the catalysis of CHS, it interacts with a number of downstream enzymes to produce a variety of flavonoid substances.

It is confirmed that the activity of CHS genes were highly regulated by plant development [19,20]. The involvement of CHS in responding against biotic factors as an easily recognized trait has also led to genetics studies of its expression in various organisms such as *Phaseolus vulgaris* [21], *Ipomea purpurea* [22] and the fungal [23]. These defensive biological factors included pathogens, bacteria and pests which promote the production of flavonoids [24–26]. Since the isolation of complete cDNA copies of CHS mRNA in cultured *Petroselinum hortense* cells [27] and a genomic clone from *Antirrhinum majus* [28], isolating CHS genes from various plants has become a hot topic, with the aim of studying their regulation through in vitro manipulation and reintroduction to plants. Through preliminary research, we speculated that *MeCHS*s could respond to the sucking stress of *T. cinnabarinus* and play a key role in the process of resisting *T. cinnabarinus*. Therefore, this study will validate the function of some *MeCHS*s as candidate anti-mite genes based on the preliminary selecting.

## Materials and methods

### Plant materials and reagents

The plant material was cassava clone ‘Xinxuan 048’ (XX048), which has been proved to be resistant to *T. cinnabarinus* infestation in previous experiments [3]. In addition, according to further analysis of proteomics and gene families, it was found that most *MeCHS* genes were up-regulated in the leaves when infested by *T. cinnabarinus* for 48 hours (12 mites per leaf). Three-month-old cassava were harvested, frozen in liquid nitrogen after infection, and stored at −80˚C until use.

Plant overexpression vector pBI121-EGFP and *Escherichia coli* DH5α were purchased from Beijing TransGen Biotech Co., LTD (Beijing, China) and Agrobacterium GV3101 was purchased from Wuhan Boyuan Biotechnology Co., LTD (Wuhan, China). Enzymes and reagents from Takara Biomedical Technology Co., LTD (Dalian, China) are as follows: PrimeScript II First Strand cDNA Synthesis kit, *Sal* I and *Spe* I restriction endonucleases, PrimerSTAR Max DNA Polymerase high fidelity Enzyme, 5×In-fusion HD Enzyme Premix; From Beijing TransGen Biotech Co., LTD (Beijing, China) are as follows: EasyPure^®^ Quick Gel Extraction kit, EasyPure^®^ Plasmid MiniPrep Kit, EasyPure^®^ Universal Plant Genomic DNA Kit, IPTG, X-gal, ampicillin (Amp), kanamycin (Kan), Rifampicin (Rif). And Silwet-77 was purchased from Coolaber Technology Co., Ltd., Beijing, China.

### RNA extraction, synthesis of cDNA strand and construction of plasmid expression vector

Total RNA was isolated from leaf samples of XX048 by using the HUAYUEYANG RNA extraction kit (Beijing, China), PrimeScript II First Strand cDNA Synthesis kit was used to synthesize cDNA for CDS sequence PCR amplification of CHS target gene coding region in cassava. pBI121-EGFP expression vector plasmid was obtained by using Plasmid MiniPrep Kit. Then the vector plasmid was double-digested with the enzyme *Sal* I (upstream) and *Spe* I (downstream). Bases on pBI121-EGFP vector sequence and the enzyme cutting sites (upstream *Sal* I and downstream *Spe* I) were combined with the specific primers of *MeCHS*s CDS sequence, and the primers were designed by Primer 5.0 software for PCR amplification of CHS gene (Table 1).

**Table 1:** Primer sequence for *MeCHS* genes clone.

Primer names	Primer sequence (5′ → 3′)	Amplicon length (bp)
*MeCHS*1-S	TCACGCCATGGTCGACATGGCTGCTGCTTCAGTAGAG	1173
*MeCHS*1-A	GCTCACCATCACTAGTTGTGGAGACACTGTGCAGCA	
*MeCHS*3-S	TCACGCCATGGTCGACATGGTGACTGTAGATGAAG	1167
*MeCHS*3-A	GCTCACCATCACTAGTAGTGGCCACGCTGTGGAG	
*MeCHS*5-S	TCACGCCATGGTCGACATGGCAGGAATAGTTGAGGAGA	1209
*MeCHS*5-A	GCTCACCATCACTAGTTACTCGATTCATTGAGTTG	

Note: The underline stands for enzyme cutting site.

The CDS sequence of *CHS* genes were cloned using the cDNA of XX048 leaves as the template, using the DNA polymerase 2×Phanta_Master_Mix (Vazyme Biotech Co., Ltd., Nanjing, China). The reaction system was shown in Table 2:

**Table 2:** The components for gene clone.

Component	Concentration	Volume (μl)
cDNA		1
upstream primers	10 μM	1
downstream primers	10 μM	1
2×Phanta_Master_Mix	2X	20
ddH_2_O		17

The PCR cycle conditions of cloned *CHS* genes were as follows: predenaturation at 95 °C for 3 min, denaturation at 95 °C for 10 s, annealing at 60 °C for 10 s, extension at 72 °C for 2 min and 30 s, 35 cycles were set from denaturation to extension, and then extension at 72 °C for 5 min, and the temperature was set at 10 °C at the end of the reaction. After completing the above setup steps, the solution is placed in the Biometra EasyCycler Gradient for reaction. The PCR products were electrophoretically detected with 1.2% agar gel, and the products containing CHS fragments were recovered with Gel recovery kit. In-Fusion HD Cloning Kit (TAKARA, Japan) was used to link the target gene to the pBI121-EGFP expression vector to obtain the PBI121-*MeCHS* vector.

### Transformation of *A. thaliana* by floral dip method

**Fig 1:**
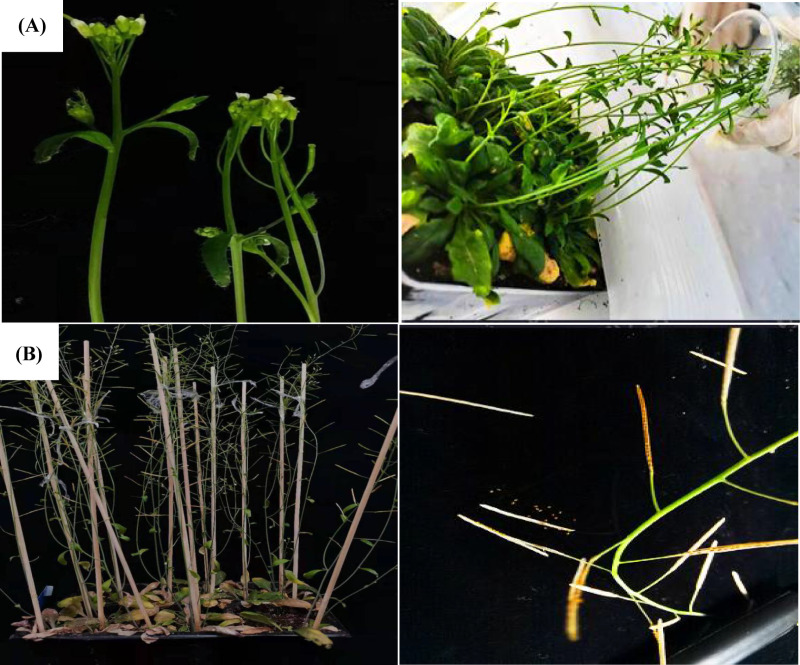
Transform *A. thaliana* inflorescence. (A) Infecting the inflorescence of *A. thaliana*; (B) Collecting seeds of *A. thaliana.*

After construction of the pBI121-*MeCHS* vector, it was transferred into *A. tumefaciens* strain GV3101. The wild-type (WT) *A. thaliana* (Columbia) plants were then transformed by the floral dip method. WT *A. thaliana* seedlings with healthy and consistent growth were selected as infection materials. The poded inflorescences were removed before the first infection, and the inflorescences were infected in the infection solution (1/2MS, 10% sucrose, 0.5μg/ml 2-morpholine-ethanesulfonic acid, 0.02% Silwet-77, PH=5.7) for 50s and then moved to the dark environment and continue cultivation for 48h. The function of the infective solution was to promote the attachment and transformation of *A. tumefaciens* into *A. thaliana.* After dark culture, the plants were transferred to normal light conditions. In order to improve the infection efficiency, after the first infection, the plant can be infected again every other week for a total of three times, and the plant should be watered normally during the infection. After the infected plants grew normally, the newly grown tidbits were cut off, and the T1 *A. thaliana* seeds were collected in time after the seeds matured, as shown in Fig 1.

### Selection and detection of transgenic *A. thaliana*

**Fig 2:**
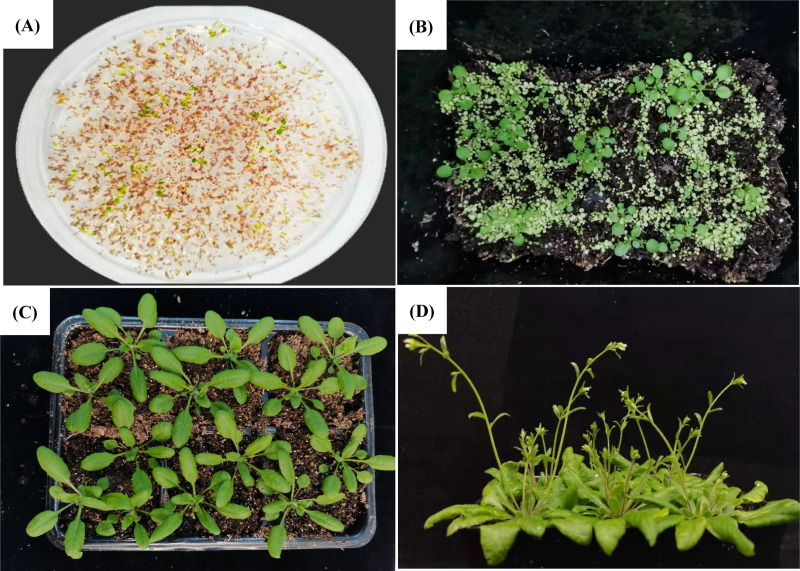
Screening transgenic *A. thaliana* plant. (A, B) Transgenic *A. thaliana* screening; (C, D) Transplantation and planting of *A. thaliana.*

The T1 generation plants were seeded in 1/2 Murashige and Skoog (MS) containing 50 mg/L kanamycin for preliminary selecting. The plants with developed roots and green true leaves were identified as T1 generation plants. The T1 generation *A. thaliana* plants were selected and cultured for 3⁓4 weeks. The leaves were extracted for detection and positive plants were screened. *A. thaliana* was cultured in the same way until T3 transgenic seeds were obtained (Fig 2).

### PCR detection of transgenic *A. thaliana*

The pBI121-*MeCHS* vector plasmid was used as positive control, and wild type *A. thaliana* leaf DNA was used as negative control. *AtActin*2 gene (NM_001338359.1) was used as a reference to detect by reverse transcription PCR, and the stable genetic homozygous positive plants of T3 generation were obtained.

### *Tetranychus cinnabarinus* rearing

**Fig 3:**
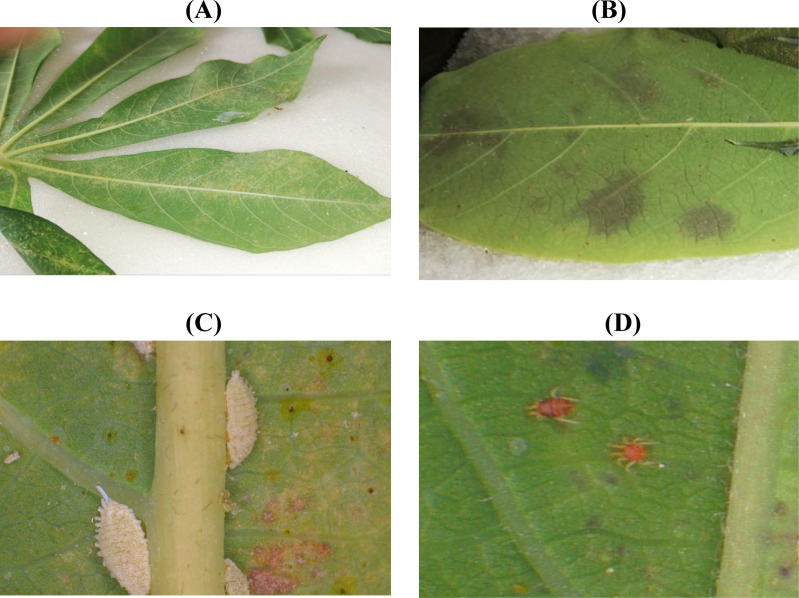
Process of artificial culture of *T. cinnabarinus.* (A) Cassava leaves on the sponge; (B) Reproduction of *T. cinnabarinus*; (C) Eggs of *T. cinnabarinus* in cassava Leaves. (D) The eggs hatch into mature *T. cinnabarinus*.

Healthy *T. cinnabarinus* adults were collected from cassava felds at Agriculture College, Guangxi University. After identifcation, the healthy and active mites were reared on the underside of fresh cassava leaves with careful observation to make sure there were no other impurities. Leaves and mites were placed on a pasteurized, water-soaked sponge and placed in glassware about 30 cm in diameter and 10 cm in height. The leaf margin was wrapped with watersaturated paper to prevent mites from escaping. Mite-infested leaves were kept under the following conditions: temperature 28±1°C, 70±5% relative humidity (RH), and 14 L:10 D photoperiod to the experiments. Fresh leaves were replaced every 2 days. The process of artificial culture of *T. cinnabarinus* was shown in Fig 3.

### Laboratory evaluation of the damage caused by *T. cinnabarinus* in transgenic *A. thaliana*

Transgenic T3 generation and wild type *A. thaliana* were cultured in pot for about 30⁓45 days. The plants were divided into infested group (10 mites per leaf with *T. cinnabarinus*) and control group (uninfested). Adult *T. cinnabarinus* were inoculated on the back of *A. thaliana* leaves in the infested group. After 24 hours, adult *T. cinnabarinus* were removed, *A. thaliana* leaves with eggs were collected. The *A. thaliana* leaves were continued to grow under conditions as *T. cinnabarinus* rearing. The following data were collected: a Number of unhatched eggs; b The number of dead mites (including juvenile mites); c Number of living mites. During the experiment, the growth and development of *T. cinnabarinus* were recorded every 24 hours, and the survival rate of F_0_ was calculated. F_0_ survival rate is calculated as follows:c/(a+b+c) [3].

### Analysis of secondary metabolites in transgenic *A. thaliana*

WT and T3 generation *A. thaliana* for 30⁓45 days were selected for the infestation treatment of *T. cinnabarinus*. Ten mites per leaf with *T. cinnabarinus* were inoculated with each *A. thaliana* for 48h, the leaves were collected and treated, and the phenotype changes were observed and recorded. At the same time, *A. thaliana* leaves cultured under normal conditions without infection were used as control. After 48h infection, the contents of tannin, flavonoids, total phenols and anthocyanins were determined.

Tannin restores phosphomolybdic acid in an alkaline environment (such as in a sodium carbonate solution), generating a blue compound. The depth of the color was directly proportional to the tannin content, and the solution had a maximum absorption peak at 760 nm. The tannin content was determined by microdetermination, and the kit purchased from Suzhou Keming Biotechnology Co., LTD., Suzhou, China. Then follow the instructions.

The determination of flavonoids in leaves includes the preparation of standard curves and the determination of samples. The production of standard curves refers to the method of Cao et al [29]. The instruments in the standard curve experiment were UV-1800 visible spectrophotometer (Shimadzu Instruments Co., Ltd., Suzhou, China) and Meilen electronic balance (Shenzhen Meifu Electronics Co., Ltd., Shenzhen, China). The sample determination method was as follows: Weigh fresh 0.2g leaves in a 10mL volumetric bottle, add 8mL of 50% ethanol solution, and extract them in a water bath at 70°C for 2 hours. The regression equation of the standard curve was as follows: Y=11.92X-0.012, R^2^=0.999, (Y was the absorbance value, X was the concentration of standard solution, and R was the correlation coefficient. The formula for calculating flavonoid content was: (Check standard curve value×dilution ratio×total volume of extract)/ sample weight×1000.

Phenols can be restored by tungstate molybdic acid under alkaline conditions to produce blue compounds, and the peak value can be determined at 760nm to obtain the total phenol content of the measured sample. A micromethod was used for the determination of Total Phenols kit, which was purchased from Suzhou Keming Biotechnology Co., LTD., Suzhou China. The determination was performed according to instructions.

Anthocyanin will react with acidic hydrochloric acid ethanol solution, making the solution a red color. The peak wavelength of anthocyanin absorption is 530nm. However, anthocyanins are interfered with by chlorophyll and soluble sugars during extraction. Therefore, in the determination process, the wavelength optical density value of the sample in chlorophyll (650nm) and soluble sugar (620nm) needs to be calculated at the same time. The optical density of anthocyanins was calculated: ODλ=(OD_530_-OD_620_)-0.1(OD_650_-OD_620_), anthocyanin content (nmol/g)=(ODλ/ε)×(V/m)×10. ODλ: Optical density of anthocyanin at 530nm wavelength;ε: molar extinction coefficient of anthocyanin 4.62×10^6^; V: total volume of extract (mL).

### Analysis of *MeCHS* genes expression in transgenic *A. thaliana*

The leaves were treated in the same way as above. Total RNA was isolated from transgenic *A. thaliana* leaves infested and uninfested by *T. cinnabarinus* samples using the HUAYUEYANG RNA extraction kit (Beijing, China). The first strand of cDNA was synthesized using TransScript® One-Step cDNA Synthesis SuperMix for qPCR (+ gDNA) from TransGen Biotech kit (Beijing, China) for *MeCHS* genes relative expression analysis, and primers were designed for qRT-PCR using primer 5.0 (Table 3). The housekeeping gene *AtActin*2 of *A. thaliana* was used as the endogenous reference gene (*AtActin*-F: AGCTATGAGTTGCCTGATGG, *AtActin*-R: ATTGTAAGTGGTCTCGTGAATAC). qRT-PCR was performed using AceQ Universal SYBR qPCR Master Mix kit (Nanjing, China). The reaction system consisted of 2.0 μl cDNA template, 0.4 μl positive and reverse primers, 10 μl 2×ChamQ SYBR qPCR Master Mix, 3.6 μL ddH_2_O. The PCR reaction instrument was QuantStudioTM 6 Flex Real Time PCR System (ThermoFisher, USA), which was programmed to predenaturate at 95°C for 3min, then react at 95°C for 10s, then react at 60°C for 30s, and then cycle 45 times. Each sample was set up with three replicates, and the relative expression level of each gene was calculated using the 2 ^− ΔΔCT^ method, where ΔΔCt = (△Ct _target gene_ - △Ct _endogenous reference gene_) treatment group - Ct (△Ct _target gene_ - △Ct _endogenous reference gene_) control group. The data were statistically analyzed with SPSS 18.0 (SPSS Science, Chicago, IL, USA) ANOVA software. Duncan’s method was used to detect the level of significant difference, and P <0.05 was considered statistically significant.

**Table 3:** The primers of *CHS* genes for qRT-PCR.

Gene Name	Forward primer sequence (5′ → 3′)	Reverse primer sequence (5′ → 3′)
*MeCHS*1	GGATATTGTAGTGGTGGAGGT	GTGAGACCCACTTCACGTAAGTGTC
*MeCHS*3	GCAAGGCTCAACGGGCGGAAG	CGATGGGCGGAGACCCAAAAG
*MeCHS*5	CACCTTATCTTCTACACAACC	CAAGTGAAAAGTGAGACCAAT

### Data analysis

ANOVA was used for statistical analysis, and SPSS 18.0 (SPSS Science, Chicago, IL, USA) was used for statistical analysis to Duncan’s tests. A value of P < 0.05 was considered a statistically significant difference. SPSS was used for correlation analysis, Pearson analysis was conducted on the mean and standard deviation of the two sample data to obtain the correlation coefficient between samples.

## Results

### Isolation *MeCHS*s and construction of expression vectors

**Fig 4:**
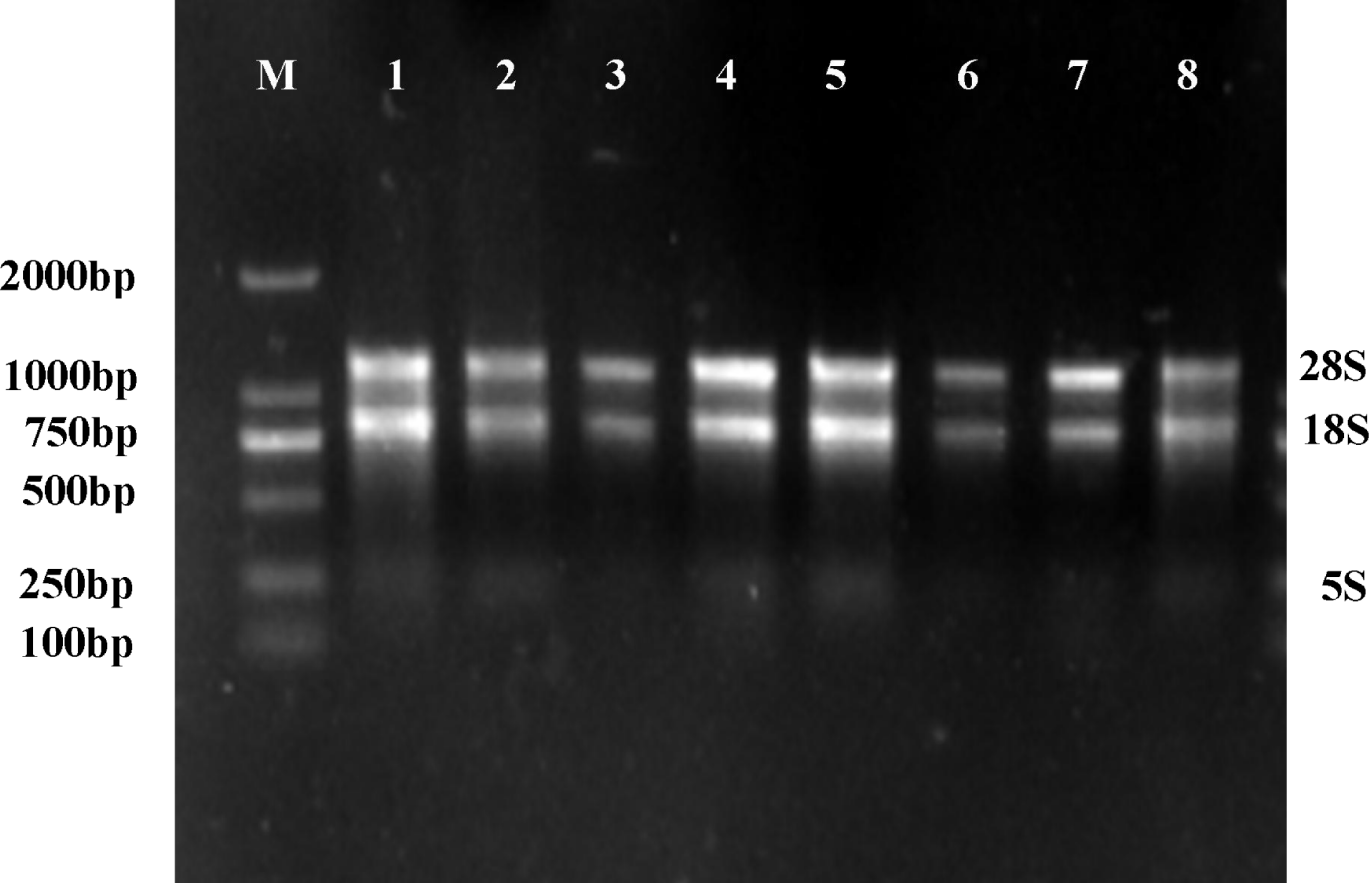
Total RNA electrophoresis of XX048 cassava leaves. Lane M: Marker DL 2000; Lanes 1-8: total RNA of XX048 cassava leaves.

RNA was extracted from the leaves of cassava genotypes XX048, and the electrophoretic detection of the extraction results was shown in Fig 4. The three rRNA electrophoresis bands of 28S, 18S and 5S in the sample were clear, and there was no obvious degradation and impurity contamination. The results showed that the total RNA concentration of the eight replicates was in the range of 460⁓900 ng/μl, and the OD_260_/OD_230_ value was in the range of 1.95⁓2.12, indicating that the integrity and purity of total RNA could meet the requirements of subsequent experimental studies.

**Fig 5:**
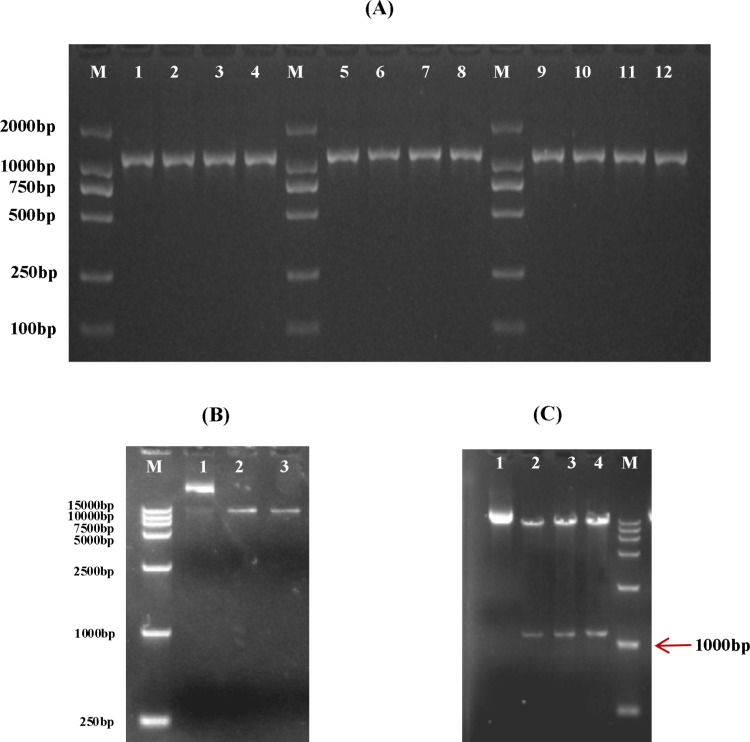
Construction of PBI121-*MeCHS* vectors. (A) PCR results of *MeCHS*1, *MeCHS*3 and *MeCHS*5 gene coding regions. Lanes 1-4: PCR of *MeCHS*1. Lanes 5-8: PCR of *MeCHS*3. Lanes 9-12: PCR of *MeCHS*5; Lane M: Marker DL 2000. (B) Double enzyme digestion of pBI121-EGFP. Lane 1: pBI121-EGFP before digestion. Lanes 2-3: pBI121-EGFP after digestion. Lane M: Marker DL15000. (C) Double enzyme digestion of recombinant plasmids PBI121-*MeCHS*. Lane 1: before digestion of plasmids. Lanes 2-4: PBI121-*MeCHS*1, PBI121-*MeCHS*3 and PBI121-*MeCHS*5 after digestion, respectively; Lane M: Marker DL15000.

cDNA was synthesized by reverse transcription using RNA as template. Then using cDNA as template, PCR amplification of CDS sequence of *CHS* genes were performed using primers in Table 1. Agarose gel electrophoresis detection results showed that a single bright band appeared in the region of about 1200 bp, and the fragment size was consistent with the expectation, as shown in Fig 5A. The expression vector pBI121-EGFP was double digested with endonuclease *Sal* I (upstream) and *Spe* I (downstream) to linearize the vector. The results were shown in Fig 5B. In-fusion ligase was used to connect the gene fragments of *MeCHS*1, *MeCHS*3 and *MeCHS*5 to the linear carrier, and the resulting ligations were transferred into *E.coli* DH5α. Then, the PBI121-*MeCHS* plasmid was double-digested by enzyme and verified by bacterial liquid PCR and sequencing. The electrophoresis results were shown in Fig 5C. Sequencing results showed that the gene product sizes of *MeCHS*1, *MeCHS*3 and *MeCHS*5 were 1176 bp, 1170 bp and 1212 bp, respectively, which were consistent with the expected gene sizes (S1 Table in S1 File). It was concluded that *MeCHS* genes had been successfully connected to the expression vector pBI121-EGFP.

### Analysis of *MeCHS* gene sequence and protein characteristics

**Fig 6:**
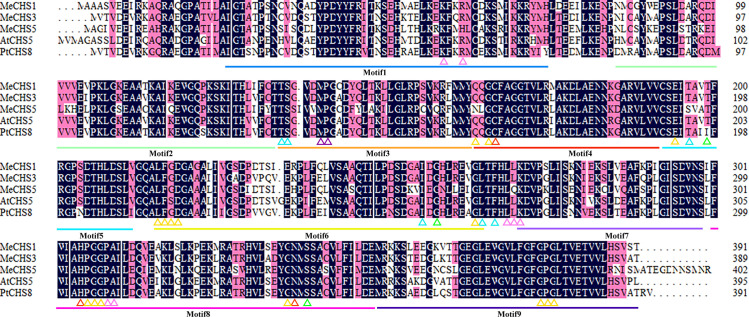
Alignment of the deduced amino acid sequence of *MeCHS*s with other plant *CHS*s. The different motifs are represented by different colors for motifs 1-9 and the structural features of the 9 motifs were listed in S3 Table in S1 File.The abbreviations for species and gene accession numbers are *Arabidopsis thaliana* CHS5 (AT5G13930.1) and *Populus trichocarpa* CHS8 (PNT46173). Catalytic residues (red), cis -peptide turn (purple), CoA binding residues (pink), and residues of the active site cavity that are structural (yellow), control polyketide size (green), or determine substrate specificity (blue) are shown in triangles.

Through previous mining of cassava transcriptomic data, three highly expressed *MeCHS*s were identified and were designated *MeCHS*1, *MeCHS*3, *MeCHS*5. The open reading frames (ORFs) of the sequences were 1176 (*MeCHS*1), 1170 (*MeCHS*3) and 1212 bp (*MeCHS*5) in length, encoding proteins of 391 (42.892 kDa), 389 (42.419 kDa) and 403 (44.786 kDa) residues, respectively. The gene sequences shared 84.14% similarity with *AtCHS*5 (accession number: AT5G13930.1) and *PtCHS*8 (accession number: PNT46173). Amino acid sequences of *MeCHS*1, *MeCHS*3, *MeCHS*5, *AtCHS*5 and *PtCHS*8 were listed in S2 Table in S1 File. Using Expasy website (https://www.expasy.org/resources/protparam) to analysis the components of *MeCHS* amino acid sequences, the three amino acids with the highest content in *MeCHS*1, *MeCHS*3 and *MeCHS*5 were Glycine Gly (G), Alanine Ala (A) and Lysine Lys (K), respectively. In addition, the content of Leu (L), Glycine Gly (G) and Alanine Ala (A), Leu (L), Serine Ser (S), Isoleucine Ile (I) were also high. The amino acid sequence comparison results of *MeCHS*s with *A. thaliana* CHS5 (AT5G13930.1) and *Populus trichocarpa* CHS8 (PNT46173) were shown in Fig 6. Besides, we found that these CHSs contained nine highly conserved domains and the structural features of the 9 motifs were listed in S3 Table in S1 File.

**Fig 7:**
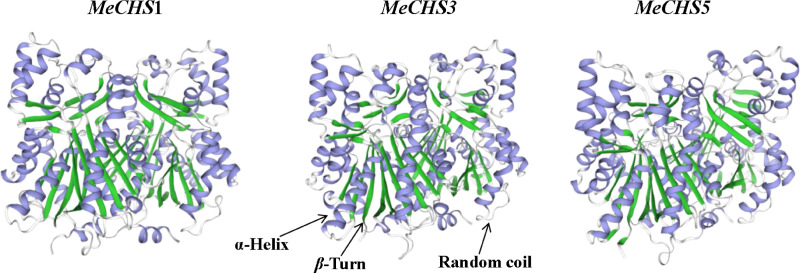
Three-dimensional structure prediction of *MeCHS* proteins.

Further to the amino acid sequence import SWISS-MODEL software (https://swissmodel.expasy.org/) for MeCHSs protein three dimensional structure prediction and analysis, the results as shown in Fig 7. As can see from the results, the protein structures of the three genes were generally similar, but there were some subtle differences. *MeCHS*1 protein has 175 α-Helixes, 123 random coils, 62 extension strands and 31 β-Turns; *MeCHS*3 protein has 177 typical α-Helixes, 135 random coils, 56 extension strands and 21 β-Turns. The *MeCHS*5 protein has 170 typical α-Helixes, 144 random coils, 64 extended strands, and 25 β-Turns.

According to the above analysis results, although the three genes all belong to *MeCHS* family, they were different in terms of morphological structure, physical and chemical properties, which may lead to one of the reasons for their functional differences.

### Expression vector transformed of *Agrobacterium*

**Fig 8:**
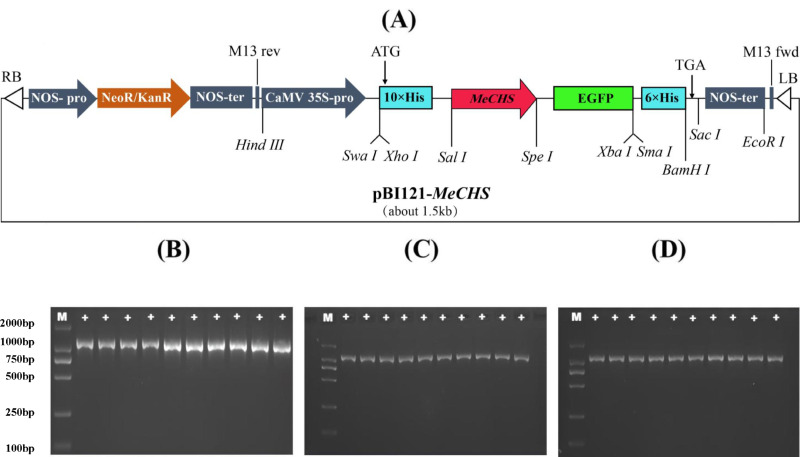
Verification of transformation of agrobacterium by expression vector PBI121-*MeCHS.* (A) Construction of PBI121-*MeCHS* vectors. (B) PCR detection of *Agrobacterium* for PBI121-*MeCHS*1; Lane M: Marker DL2000. (C) PCR detection of *Agrobacterium* for PBI121-*MeCHS*3; Lane M: Marker DL2000. (D) PCR detection of *Agrobacterium* for PBI121-*MeCHS*5; Lane M: Marker DL2000. +: positive of *Agrobacterium* liquid.

The obtained recombinant PBI121-*MeCHS* carrier construction was shown in Fig 8A, and the recombinant plasmid were transformed into *Agrobacterium* GV3101. As a regulatory element, the 35S promoter from the plant pathogen Cauliflower Mosaic Virus (CaMV) has been instrumental in driving constitutive expression of these transgenes. Subsequently, PCR was performed to verify the bacterial solution, and the results were shown in Fig 8B–D.

### Screening and identification of transgenic *A. thaliana*

**Fig 9:**
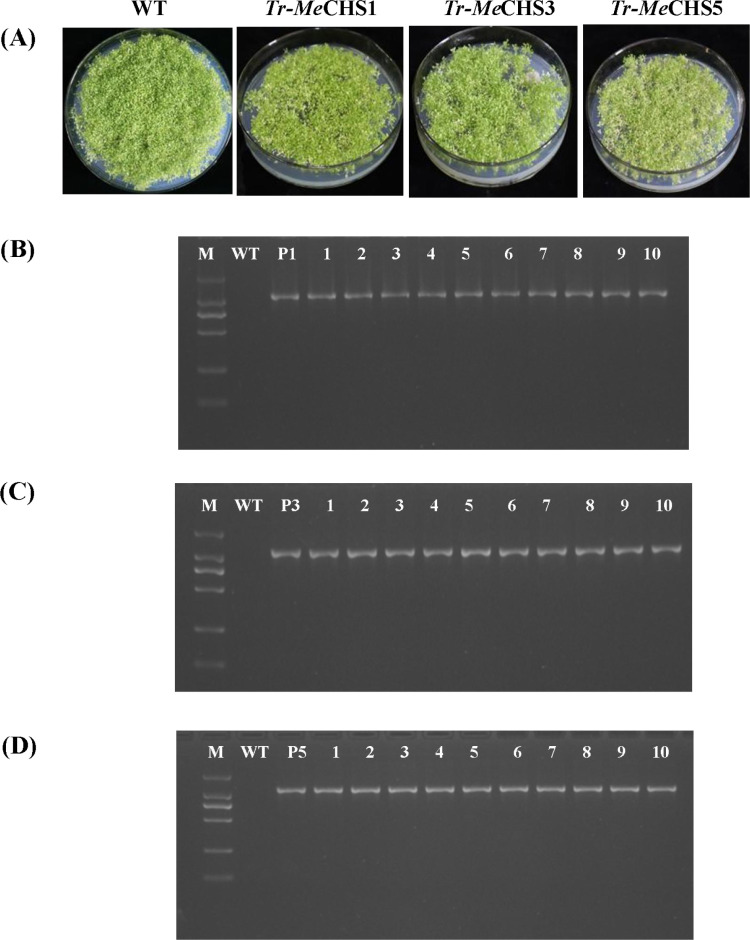
Selection and identification of transgenic *A. thaliana.* (A) Resistance Selection of T3 generation seeds from *A. thaliana*. (B) PCR of transgenic *A. thaliana* with *MeCHS*1. Lanes P1: positive control (pBI121-*MeCHS*1). Lanes 1-10: transgenic *A. thaliana* with *MeCHS*1. (C) PCR of transgenic *A. thaliana* with *MeCHS*3. Lanes P3: positive control (pBI121-*MeCHS*3). Lanes 1-10: transgenic *A. thaliana* with *MeCHS*3. (D) PCR of transgenic *A. thaliana* with *MeCHS*5. Lanes P5: positive control (pBI121-*MeCHS*5). Lanes 1-10: transgenic *A. thaliana* with *MeCHS*5. Lane M: Marker DL 2000. WT: wild type.

After insertion of the cDNA coding sequence into the pBI121 vector to generate an overexpression construct PBI121-*MeCHS* and transformation with *Agrobacterium* GV3101, lines stably overexpressing *MeCHS*1, *MeCHS*3, *MeCHS*5 were generated in the Columbia-0 background of *A. thaliana*. Transgenic *A.thaliana* T3 seeds were screened in the culture medium containing antibiotics, and the results were shown in Fig 9. Wild type *A.thaliana* (WT) had all green leaves, while transgenic *A.thaliana* (*Tr-MeCHS*1, *Tr-MeCHS*3 and *Tr-MeCHS*5) showed albino seedlings in screening media. Those with green leaves were positive *A.thaliana* transferred to the target gene, as shown in Fig 9A. About 40 days after transplanting and culture of these *A.thaliana* seedlings, DNA was extracted and then RT-PCR was performed. As shown in Fig 9B–D, more than 10 strains of each transgenic line could successfully amplified specific bands consistent with the expected fragment size of *MeCHS*s, while the wild type did not have any bands, indicating that *MeCHS*s could be stably expressed at the transcriptional level of transgenic *A.thaliana*. These results proved that *MeCHS*s have been successfully transferred into *A.thaliana* and can be stably inherited, which can be used for further gene verification analysis.

### Indoor identification of *T. cinnabarinus* resistance of transgenic *A. thaliana*

**Fig 10:**
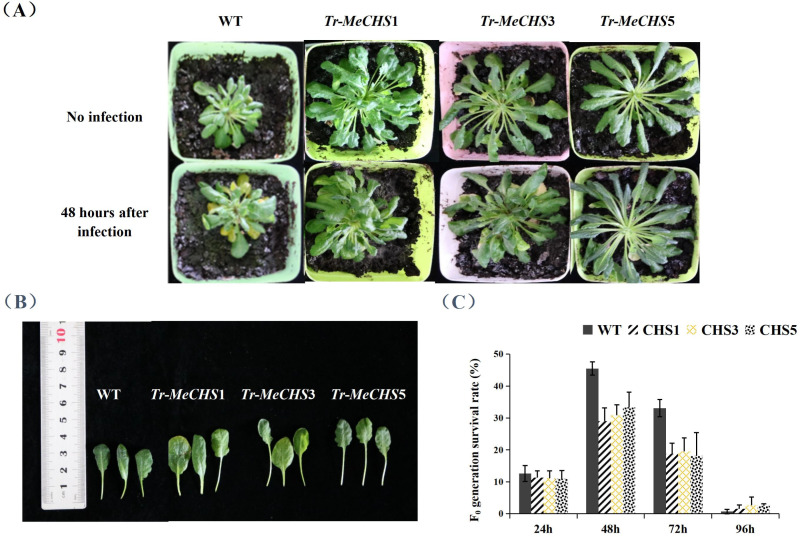
Indoor identification of *T. cinnabarinus* resistance of transgenic *A. thaliana.* (A) Comparison of *A. thaliana* infested by *T. cinnabarinus*. (B) Leaf comparison of *A. thaliana*. (C) F_0_ generation survival rate of *T. cinnabarinus* in *A. thaliana.* WT represented wild *A. thaliana*, *Tr-CHS*1, *Tr-CHS*3, *Tr-CHS*5 represented transgenic *A. thaliana* with *MeCHS*1, *MeCHS*3 and *MeCHS*5 respectively.

The experimental materials were wild type (WT) *A.thaliana* and transgenic *A.thaliana* plants (*Tr-MeCHS*1, *Tr-MeCHS*3 and *Tr-MeCHS*5). Wild type and transgenic *A. thaliana* were infected with 10 *T. cinnabarinus* per leaf, and the adult *T. cinnabarinus* were removed 24 hours later. The growth and development of the *T. cinnabarinus* were recorded, and the F_0_ survival rate was calculated. The results showed that the F_0_ generation experienced about 4 days from egg to adult *T. cinnabarinus*, and at 48h after leaf infection, the growth number of *T. cinnabarinus* reached the highest value both wild and transgenic *A. thaliana* (Fig 10A, B). At that time, the survival rate of F_0_ in WT, *Tr-MeCHS*1, *Tr-MeCHS*3 and *Tr-MeCHS*5 leaves were 45.49%, 29.03%, 30.84% and 33.33%, respectively (Fig 10C). Compared with WT, the F_0_ survival rate of *T. cinnabarinus* in transgenic *A. thaliana* were reduced by 36.18% in *Tr-MeCHS*1, 32.20% in *Tr-MeCHS*3 and 26.73% in *Tr-MeCHS*5. The results showed that the survival rate of *T. cinnabarinus* in transgenic lines were lower than that in wild-type lines, and the difference between them reached a significant level, indicating that the transfer of *MeCHS* genes were beneficial to control the further reproduction of *T. cinnabarinus*.

### Comparison of secondary metabolite contents between wild-type and transgenic *A. thaliana*

**Fig 11:**
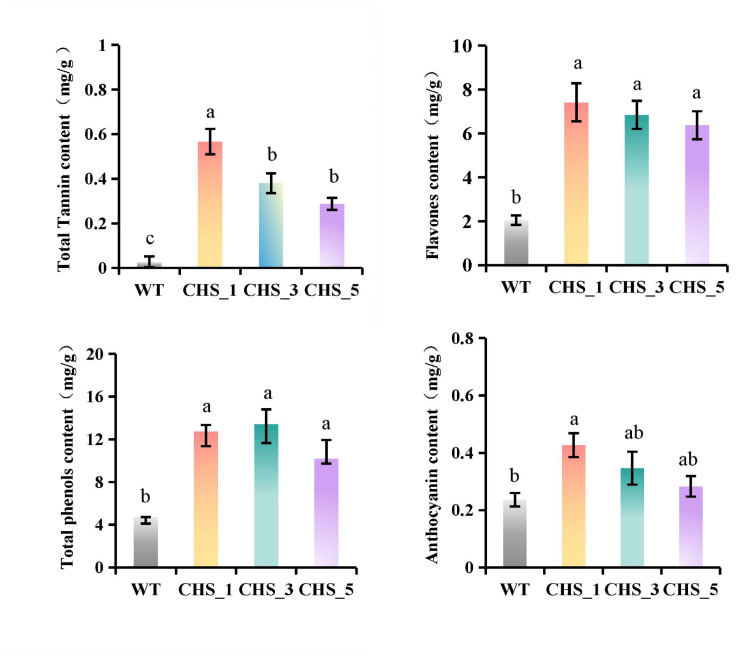
Comparison of tannins, flavonoids, total phenols and anthocyanins contents between wild-type and transgenic *A. thaliana.* WT represented wild *A. thaliana*, CHS_1, CHS_3, CHS_5 represented transgenic *A. thaliana* with *MeCHS*1, *MeCHS*3 and *MeCHS*5 respectively. The raw data were listed in S4 Table in S1 File. These values are presented as the mean±standard deviation (SD) based on three biologically independent values. Letters above the histogram indicate the statistical signifcance (p<0.05) and bars with the same letter are not significantly different from each other. The same below.

The contents of tannin, flavonoids, total phenols and anthocyanins of transgenic and wild *A. thaliana* were determined respectively, and the results were shown in Fig 11. Compared with the WT, the tannin activity of *Tr-MeCHS*1, *Tr-MeCHS*3 and *Tr-MeCHS*5 *A. thaliana* increased 20.25 times, 13.25 times and 9.75 times, respectively. The above values were extremely significantly different (P<0.01). In transgenic *A. thaliana* the flavonoid content was increased by 2.61 times (*Tr-MeCHS*1), 2.33 times (*Tr-MeCHS*3) and 2.10 (*Tr-MeCHS*5) times respectively, and the values were extremely significantly different compared with the WT *A. thaliana*. The total phenol content increased by 1.69 times (*Tr-MeCHS*1), 1.83 times (*Tr-MeCHS*3) and 1.15 times (*Tr-MeCHS*5), respectively, and the values were significantly different (P<0.05) compared with the WT lines. The anthocyanin content was increased by 80.26% (*Tr-MeCHS*1, significantly different compared with WT), 46.46% (*Tr-MeCHS*3, no significant difference compared with WT) and 19.70% (*Tr-MeCHS*5, no significant difference compared with WT), respectively.

### Analysis of *MeCHS* gene relative expression in *A. thaliana*

**Fig 12:**
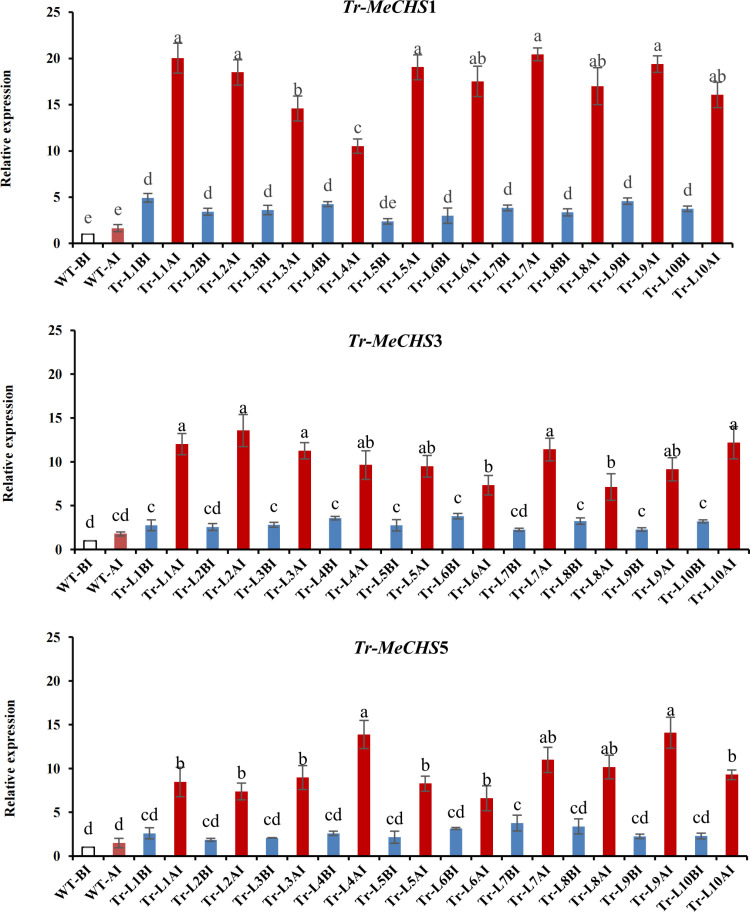
Analysis of *MeCHS* genes relative expression in *A. thaliana.* WT represented wild *A. thaliana*, Tr represented transgenic *A. thaliana*, BI represented before infection, AI represented after infection. The raw data were listed in S5 Table in S1 File.

Wild type and transgenic *A. thaliana* were infected with 10 *T. cinnabarinus* per leaf, and the uninfected group was used as the control. After 48 hours of infection, the CHS genes expressions were measured. As shown in Fig 12, compared with uninfected lines, *MeCHS*s expression levels of *A. thaliana* were increased under the sucking stress of *T. cinnabarinus*. These results indicated that *T. cinnabarinus* could induce the increase of CHS genes expression. Among the *A. thaliana* plants (10 experimental replicates) the average gene expression of *Tr-MeCHS*1 before infection was 3.71 and after infection was 17.31, in which the gene expression of L7 and L1 strains of *Tr-MeCHS*1 was 12.38 times and 12.14 times compared with non-transgenic *A. thaliana*, respectively. The average gene expression of *Tr-MeCHS*3 was 2.93 before infection and 10.31 after infection. The expression of L2 and L10 of *Tr-MeCHS*3 were 7.67 times and 6.88 times compared with non-transgenic *A. thaliana*, respectively. The average gene expression of *Tr-MeCHS*5 was 2.61 before infection and 9.80 after infection. The L9 and L4 of *Tr-MeCHS*5 had high levels of gene expression, which were 9.45 times and 9.31 times compared with WT lines, respectively.

### The correlation analysis of *MeCHS*s expression level with secondary metabolites and survival rate of *T. cinnabarinus*

The correlation analysis of *MeCHS* genes expression with secondary metabolites and survival rate of *T. cinnabarinus* were shown in Table 4. According to the data, the expression level of *MeCHS*s were extremely significant positive correlation with the content tannin and flavonoid, significant positive correlation with total phenol and anthocyanin content, and negative correlation with F_0_ survival rate of *T. cinnabarinus*. The F_0_ survival rate of *T. cinnabarinus* were extremely significantly negatively correlated with flavonoid and total phenol content, significantly negatively correlated with tannin and anthocyanin content. According to the above analysis, the genes expression of *MeCHS* were positively correlated with the synthesis of secondary metabolites, and negatively correlated with the F_0_ survival rate of *T. cinnabarinus*. These results indicated that the different expression levels of *MeCHS* genes lead to different content of secondary metabolites, and finally the *A. thaliana* had different resistance to *T. cinnabarinus*.

**Table 4:** The correlation analysis of *MeCHS*s expression levels with secondary metabolites and survival rate of *T. cinnabarinus.*

	X1	X2	X3	X4	X5	X6
**X1**	1					
**X2**	0.822**	1				
**X3**	0.831**	0.779*	1			
**X4**	0.796*	0.832**	0.916**	1		
**X5**	0.706*	0.635	0.846**	0.630	1	
**X6**	-0.740*	-0.783*	-0.927**	-0.962**	-0.678*	1

Note: * and ** mean significant at p< 0.05 and extremely significant at p< 0.01. X1-X6 represent *MeCHS*s expression levels, tannin content, flavonoid content, total phenol content, anthocyanin content and F_0_ survival rate of *T. cinnabarinus*, respectively.

## Discussion

### *MeCHS*s affected the synthesis and accumulation of secondary metabolites

As the first key enzyme and rate-limiting enzyme in the biosynthesis of secondary metabolites, CHS is located in the upstream of flavonoid biosynthesis pathway and can synthesize the basic skeleton of flavonoid chalcone [30]. Chalcone not only provides the basic carbon skeleton structure for flavonoid substances, but also provides the basic guarantee for the synthesis of secondary metabolites such as isoflavones, flavonoids, tannins and anthocyanins. More importantly, CHS can regulate the synthesis and accumulation of secondary metabolites on other phenylalanine branches through substrate competition.

In this study, the *A. thaliana* with heterogeneously expressing *MeCHS*1, *MeCHS*3 and *MeCHS*5 genes were infected by *T. cinnabarinus*, and it was found that the expression level of *MeCHS*s in all transgenic lines were significantly increased, and the content of secondary metabolites were also increased. Pang found a significant positive correlation between CHS gene and flavonoid content in Ginkgo biloba [31]. Wang cloned *CitCHS*1, *CitCHS*2 and *CitCHS*3 genes of citrus and transferred them into tobacco or citrus, and found that the production of total flavonoids was regulated by the overall expression of CHS family genes [32]. The results of this study were similar to those of the above experiments, indicating that the synthesis process of secondary metabolites was positively regulated by *MeCHS*s expression.

### *MeCHS*s can improve the resistance of plants to biotic stress

Since 1983, with the first complete cDNA copy of *CHS* gene mRNA isolated from cultured *Petroselinum hortens* cells, nearly 20 functionally distinct *CHS* superfamilies have been found and further identified [27,33]. In recent years, there have been a large number of reports related to *CHS*, indicating that *CHS* exists widely in plants and plays an important role in many physiological and biochemical activities. *CHS* not only has a variety of biological functions in pigment synthesis, resistance to ultraviolet radiation [4,5,34], resistance to stress, regulation of auxin transport and pollen fertility during growth and development [8,35–37], but also plays an important role in preventing plants from being harmed by various pathogens and insect resistance [38]. Several studies have shown that *CHS* has strong tissue-specific expression when regulated by different factors, such as in response to biological factors such as fungi, pathogens, bacteria, and pest defense. *CHS* is also significantly associated with the synthesis of flavonoid compounds, and these biological factors promote the production of flavonoids [25,39,40]. Lawton showed that the expression level of *CHS* in bean cells was significantly increased after being treated with anthrax extract for 20min, and reached the highest value after being treated for 2.5 h, indicating that some activators in anthrax extract induced the expression of *CHS* gene in beans [41]. Ran found that the emergence of *CHS*V and *CHS*VII was significant for the pathogenicity development of pathogenic fungi [23]. The *CHS* mutant was severely damaged by herbivorous insects and seriously infected by *Rhizoctonia solani* [24]. Studies have shown that *CHS* genes were closely related to plant resistance and secondary metabolite synthesis. Different inducible factors increased the expression of *CHS*, and then promoted the production of flavonoids and other related secondary metabolites. These substances played a vital role in plant resistance as antibiotics or ingestion inhibitors. Although some *CHS* genes have been reported in plants such as *A. thaliana*, citrus and beans, less research has been done on the *CHS* genes in cassava, only its drought resistance has been studied.

In this study, the genes *MeCHS*1, *MeCHS*3 and *MeCHS*5 were transferred into *A. thaliana*, and then were infected by *T. cinnabarinus*. The results showed that compared with non-transgenic *A. thaliana*, the F_0_ survival rate of *T. cinnabarinus* in *Tr-MeCHS*1 decreased by 36.18%, *Tr-MeCHS*3 by 32.20% and *Tr-MeCHS*5 by 26.73%. The expression of genes also affects the morphology of leaves. As shown in Fig 10B, the leaves exhibited yellowing after infection. In our experiment, *Arabidopsis* plants transferred to *MeCHS* not only inhibited *T. cinnabarinus* reproduction but also had larger leaves compared with non-transgenic controls. We hypothesize that this enhanced growth was attributable to reduced *T. cinnabarinus* infestation, which in turn promotes healthier plant development. These results showed that heterologous transformation of *MeCHS*1, *MeCHS*3 and *MeCHS*5 genes from *Manihot esculenta* Crantz lead to the synthesis of secondary metabolites in *A. thaliana* which may protect against *T. cinnabarinu*s.

### The expression activity of *MeCHS*s family members were different

Spatiotemporal differences in the expression of CHS gene family led to their different contributions to flavonoid biosynthesis. CHS gene expression in transgenic *A. thaliana* plants increased under piercing-sucking stress, and the difference between transgenic lines and non-transgenic lines reached an extremely significant level. However, from the experimental results, the expression levels of the three transgenic plants were also inconsistent. Obviously, the expression level of *Tr-MeCHS*1 gene was higher than the other two *CHS* gene family members.

Except in blank control plants, the content of secondary metabolites increased significantly in *A. thaliana* plants with CHS gene. By the biosynthesis determination of the tannins, flavonoids, total phenols and anthocyanins, it was found that *A. thaliana* plants transferred to *MeCHS*1 gene had higher expression and higher secondary metabolites. Further study showed that the F_0_ survival rate of *T. cinnabarinus* in *A. thaliana* was reduced to different extent by different *MeCHS* genes. Correlation analysis showed that the expression of *MeCHS* genes was positively correlated with the synthesis of secondary metabolites, and negatively correlated with F_0_ survival rate of *T. cinnabarinus*. These results indicated that the different expression levels of *MeCHS*s lead to the difference of secondary metabolites, and thus the resistance of *A. thaliana* plants to *T. cinnabarinus* was also different.

Periyasamy reported that CHS genes have different expression patterns, and the study found that the expression activity of two different CHS genes is very different [42]. In recent years, Wang [32] found that the accumulation and total expression levels of three CHS genes and flavonoids in citrus were different, and the correlation coefficients between *CitCHS*1, *CitCHS*2 and *CitCHS*3 and flavonoids were 0.90, 0.43 and 0.80, respectively. Further studies showed that the biosynthesis and reduction of flavonoids could be achieved by controlling the up-regulated or down-regulated expression of the CHS genes. The above results were similar to our experiment.

The study on CHS family genes showed that these genes have some commonalities, such as structural similarity and sequence conservation. The transferred *MeCHS*s showed similar functions, improving the biosynthesis of flavonoids, and thus inhibiting the reproduction of *T. cinnabarinus*. However, the expression difference between different members and the internal regulatory mechanism of flavonoid interaction between *MeCHS* genes still need to be further explored and analyzed.

## Conclusion

To verify the gene function of *MeCHS*1, *MeCHS*3 and *MeCHS*5, we constructed plant expression vectors and then heterogeneously expressing them in *A. thaliana*. Results displayed that the *T. cinnabarinus* F_0_ survival rate of *A. thaliana* reached the highest value after inoculation in their leaves for 48h. However, it decreased in transgenic *A. thaliana*, and the difference between the transgenic and wild type reached a significant level, indicating that overexpression of CHS genes were conducive to controlling the further reproduction of *T. cinnabarinus*. Meanwhile, *MeCHS*1, *MeCHS*3 and *MeCHS*5 genes could promote *A. thaliana* to synthesize more secondary metabolites such as tannins, flavonoids, total phenols and anthocyanins. The results of gene expression analysis showed that *Tr-MeCHS*1 had the highest expression level among the three genes, indicating that the expression levels of different *MeCHS*s family members were different. Correlation analysis showed that the expression of *MeCHS*s were positively correlated with the synthesis of secondary metabolites, and negatively correlated with F_0_ survival rate of *T. cinnabarinus*. In conclusion, the different expression levels of *MeCHS* genes led to the difference in the synthesis of secondary metabolites, and further affected the resistance of *A. thaliana* to *T. cinnabarinus*. This study is of great significance for saving the serious economic loss caused by *T. cinnabarinus* infestation on cassava. These data will further deepen our understanding both the molecular mechanisms and role of *MeCHS* genes. In addition, the results will help to further elucidate the effects on *T. cinnabarinus* and provide a theoretical basis for the potential functions of the specific CHS gene in resistance to mites and other biotic stress.

## Supporting information

S1 File **S1 Table.** cDNA sequences of *MeCHS*1, *MeCHS*3 and *MeCHS*5 in cassava. **S2 Table.** Amino acid sequences of *MeCHS*1, *MeCHS*3, *MeCHS*5, *AtCHS*5 and *PtCHS*8. **S3 Table.** Conserved motifs of *MeCHS*s. **S4 Table.** Comparison of tannins, flavonoids, total phenols and anthocyanins contents between wild-type and transgenic *Arabidopsis thaliana*. **S5 Table.** Analysis of *MeCHS* gene relative expression in *Arabidopsis thaliana*.
(XLSX)

S1 Fig Flavonoid biosynthesis pathway. (DOCX)
