# Supplementary material for: Molecular cloning and functional verification of chalcone synthase genes from cassava (Manihot esculenta Crantz) in defense against Tetranychus cinnabarinus infestation
Source: PLoS One. 2025 Apr 24;20(4):e0321276. doi: 10.1371/journal.pone.0321276 (PMC12021200; doi:10.1371/journal.pone.0321276)
Supplement: S1 Fig — (DOCX) [file pone.0321276.s002.docx]

Supplementary Figure

Figure S1 Flavonoid biosynthesis pathway
